# Case report: Combined therapy of bilateral subthalamic nucleus deep brain stimulation and spinal cord stimulation significantly improves motor function in a patient with multiple system atrophy with predominant parkinsonism

**DOI:** 10.3389/fnins.2022.929273

**Published:** 2022-08-01

**Authors:** Jiping Li, Shanshan Mei, Xiaohua Zhang, Yunpeng Wang, Xiaofei Jia, Jinlong Liu, Erhe Xu, Wei Mao, Yuqing Zhang

**Affiliations:** ^1^Department of Functional Neurosurgery, Beijing Institute of Functional Neurosurgery, Xuanwu Hospital, Capital Medical University, Beijing, China; ^2^Department of Neurology, Xuanwu Hospital, Capital Medical University, Beijing, China

**Keywords:** multiple system atrophy, deep brain stimulation, spinal cord stimulation, subthalamic nucleus, freezing of gait

## Abstract

Multiple system atrophy with predominant parkinsonism (MSA-P) is a highly incapacitating disease with a short life expectancy and symptomatic therapy is still limited. In this report, we presented the case of a 65-year-old woman with a 3-year history of severe rigidity, bradykinesia, and gait dysfunction alongside severe freezing of gait diagnosed with MSA-P. She underwent combined therapy of bilateral subthalamic nucleus deep brain stimulation (DBS) and low-thoracic spinal cord stimulation (SCS). The double-blind evaluation of the Movement Disorder Society Sponsored Revision of the Unified Parkinson’s Disease Rating Scale part III and 7-m Timed Up and Go at follow-ups showed her cardinal parkinsonian symptoms benefit significantly from DBS stimulation, while the improvement of SCS was mainly embodied in lower-limb symptoms. The combined stimulation achieved a better improvement of motor function than either DBS or SCS stimulation alone. Most notably, the improvement of lower-limb symptoms was significantly enhanced by the combined stimulation.

## Introduction

Multiple system atrophy with predominant parkinsonism (MSA-P) presents with parkinsonian symptoms as its prominent manifestation and may have limited response to levodopa. It is a highly incapacitating disease with a mean survival of 6–10 years (O’Sullivan et al., 2008; Wenning et al., 2013), and symptomatic therapy is still limited (Meissner et al., 2019). Deep brain stimulation (DBS) is generally not recommended for patients with MSA, despite DBS having benefited to some variable extent (Visser-Vandewalle et al., 2003; Huang et al., 2005; Kim et al., 2012; Ullman et al., 2012; Zhu et al., 2014; Meissner et al., 2016), and the clinical improvement was short-lasting and rapidly followed by the early onset of gait dysfunction or postural instability with falls, which were not improved by DBS (Talmant et al., 2006; Meissner et al., 2016). Recently, some pilot studies demonstrated that spinal cord stimulation (SCS) improved gait problems in patients with Parkinson’s disease (PD) (Agari and Date, 2012; de Andrade et al., 2016; de Souza et al., 2017; Samotus et al., 2018, 2020a), MSA-P (Zhang et al., 2020) or progressive supranuclear palsy (PSP) (Samotus et al., 2020b). Here, we reported the combined therapy of bilateral subthalamic nucleus (STN) DBS and low-thoracic SCS significantly improves motor function in a patient with MSA-P with severe rigidity, bradykinesia, and gait dysfunction alongside severe freezing of gait (FOG).

## Case report

The patient noticed tremor and gradual loss of dexterity in her right limbs in 2017 at the age of 62 years. Since 2018, she had difficulty turning over on the bed, and levodopa therapy was initiated but was unhelpful. Within 2 years, she developed left-hand tremor and started walking with small steps, had slow turning, freezing, and occasional falls. Since 2020, she had difficulty arising from a chair and was barely able to walk, and her symptoms were not improved with levodopa treatment. In July 2020, the patient was admitted to our hospital with complaints of severe akinetic rigidity and difficulty walking.

**FIGURE 1:**
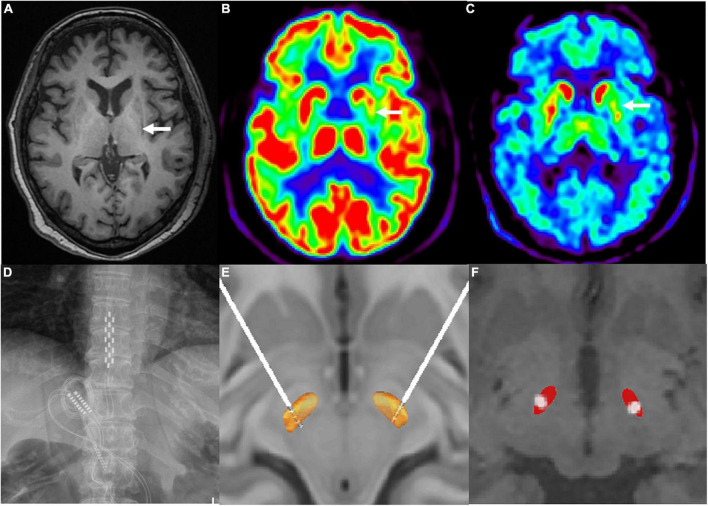
**(A)** Left hypointense lateral putaminal rim on T1-signal cranial magnetic resonance imaging (as indicated by the white arrow). **(B)** ^18^F-fluorodeoxyglucose positron emission tomography scan revealed reduced in glucose metabolisms in the left putamen (as indicated by the white arrow). **(C)** ^18^F-fluoropropyl-(+)-dihydrotetrabenazine positron emission tomography scan revealed hypometabolism in the left putamen (as indicated by the white arrow). **(D)** paddle-shaped electrode of spinal cord stimulation implanted into the epidural space at T10–T12 spinal cord segments. **(E)** 3D illustration for the localization of deep brain stimulation electrode contacts (model 3389, Medtronic) by the lead DBS software. Electrodes were implanted as follows: contacts 0, 1, and 2 were located inside the STN and contact 3 was located above the STN. **(F)** 2D illustration for the localization of active contacts by the lead DBS software.

On neurological examination, she displayed severe bradykinesia and rigidity, and moderate postural and resting tremors in the limbs, severe FOG, and spontaneous falls. Pull testing was positive, Romberg sign was negative, oculomotor examinations were normal, and she showed no finger-to-nose dysmetria or heel-to-shin ataxia. Neuropsychological studies ruled out dementia or major depression: the score of Mini-Mental State Examination (MMSE) was 27, Montreal Cognitive Assessment (MoCA) was 24, Hamilton Anxiety Scale (HAMA) was 15, and Hamilton Depression Scale (HAMD) was 18. The residual urine volume was 98.6 ml, supine blood pressure was 132/87 mmHg, and 100/65 mmHg after standing for 3 min. In the acute levodopa challenge test, the improvement in the Movement Disorder Society Sponsored Revision of the Unified Parkinson’s Disease Rating Scale Part III (MDS-UPDRS-III) motor score was 11% (91 vs. 81) without obvious on and off statements. Preoperative brain magnetic resonance imaging (MRI) revealed mild cerebral atrophy and a left lateral putaminal rim (Figure 1A). ^18^F-fluorodeoxyglucose-positron emission tomography (FDG-PET) showed hypometabolism mainly in the left putamen, and ^18^F-fluoropropyl-dihydrotetrabenazine PET revealed that vesicular monoamine transporter type 2 (VMAT2) distribution was decreased mainly in the left putamen (Figures 1B,C). Clinically probable MSA-P was diagnosed based on the diagnostic scheme for MSA (Palma et al., 2018).

To release her akinetic rigidity and gait-related problems simultaneously, combined DBS and SCS electrode implantation was performed in August 2020 after a full explanation of potential risks and benefits. A paddle-shaped SCS electrode (Adaptive Stim^®^ 39565; Medtronic, Minneapolis, MN) was implanted into the epidural space at thoracic levels ranging from the T10 to T12 spinal segments (Figure 1D). DBS electrodes (Activa^®^ 3389, Medtronic, Minneapolis, MN) were implanted into the bilateral STN. Intraoperative local field potential recordings showed elevated beta activity (frequency at 21–30 Hz) in the bilateral STN (Supplementary Figure 1). Postoperative CT images were fused with the preoperative MRI images to confirm the coordinates of the DBS electrode metal tip relative to the midcommissural point were left (X, Y, Z –9.5, –4.03, –7.65 mm) and right (10.05, –3.32, –7.56 mm). Figures 1E,F show the localization of electrode contacts and the active contacts by the lead DBS software.

**FIGURE 2:**
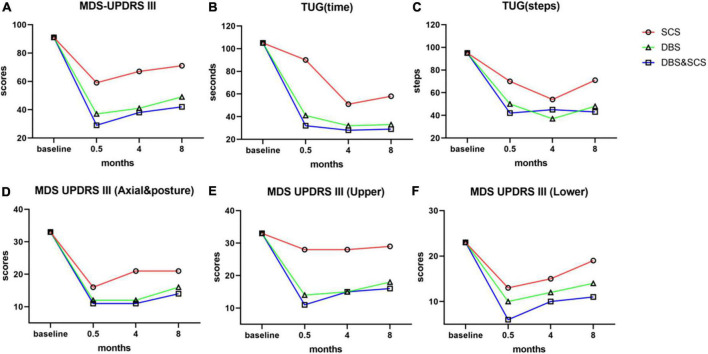
The outcomes of MDS-UPDRS III and TUG in the medication-off state prior to surgery and at 0.5- (phase I trials), 4-, and 8-month postoperative. The stimulation test was performed separately, and the outcome was evaluated double-blindly at all follow-ups. **(A–C)** Outcome of total scores of MDS-UPDRS III **(A)**, time **(B)**, and steps **(C)** of the 7-m TUG by SCS stimulation (red lines), DBS stimulation (green lines), and DBS and SCS stimulation (blue lines). The combined stimulation of DBS and SCS showed a better improvement in MDS-UPDRS III and TUG than either SCS or DBS stimulation. **(D–F)** Subscores of MDS-UPDRS III, axial and posture subscores **(D)**, upper-limb subscores **(E)**, and lower-limb subscores **(F)** of MDS-UPDRS III. Axial and posture subscores: items 3.1, 3.2, 3.3, 3.9, 3.10, 3.11, 3.12, 3.13, and 3.14; upper-limb subscores: items 3.3, 3.4, 3.5, 3.6, 3.15, 3.16, and 3.17; lower-limb subscores: items 3.3, 3.7, 3.8, and 3.17. DBS stimulation and DBS and SCS stimulation did significantly improve the cardinal parkinsonian symptoms, and the improvement of upper limbs and axial symptoms mainly benefits from DBS stimulation, SCS stimulation alone slightly improves the cardinal parkinsonian symptoms, and the improvement was mainly embodied in lower limbs and axial symptom; most notably, the improvement of lower-limb symptoms was significantly enhanced by the combined stimulation. MDS-UPDRS III, Movement Disorder Society Sponsored Revision of the Unified Parkinson’s Disease Rating Scale part III; TUG, Timed Up and Go test; SCS, spinal cord stimulation; DBS, deep brain stimulation.

The electrodes and the impulse generators were implanted in two stages separated by a 15-day double-blind phase I trials session (Supplementary Figure 2), which was held using external stimulator, the MDS-UPDRS III, the Unified Multiple Systems Atrophy Rating Scale Part I, II, IV (UMSARS I, II, IV), and 7-m Timed Up and Go (TUG) test were evaluated double-blindly in med-off condition. The combined stimulation showed a better improvement than either DBS or SCS stimulation alone (Table 1 and Figure 2). Finally, pulse generators were implanted.

**TABLE 1:** The outcomes of the double-blind evaluation of the MDS-UPDRS III, UMSARS I, II, IV, and TUG held at baseline and 0.5- (phase I trials), 4-, and 8-month postoperative.

	Baseline	0.5-month			4-month			8-month		
		SCS	DBS	DBS&SCS	SCS	DBS	DBS&SCS	SCS	DBS	DBS&SCS
**MDS-UPDRS III**										
Rigidity (3.1-3.3item)	22	15	11	8	17	10	6	18	10	6
Upper score	6	5	3	2	5	3	2	5	0	0
Lower score	6	5	4	2	4	3	1	5	4	2
Axial score	10	5	4	4	8	4	3	8	6	4
Bradykinesia (3.4-3.8item)	33	23	15	11	27	21	22	32	23	22
Upper score	18	15	9	7	16	12	13	18	13	13
Lower score	15	8	6	4	11	9	9	14	10	9
Tremor (3.15-3.18item)	13	10	3	3	10	2	2	8	6	4
Upper score	9	8	2	2	7	2	2	6	5	3
Lower score	2	0	0	0	0	0	0	0	0	0
Posture (3.9-3.14item)	23	11	8	7	13	8	8	13	10	10
3.9 arising from chair	4	2	1	1	2	1	1	1	1	1
3.10 gait	4	2	1	1	2	2	2	2	1	1
3.11freezing of gait	4	0	0	0	0	0	0	1	1	1
3.12 postural stability	4	2	2	2	3	3	3	3	3	3
3.13 posture	3	2	2	2	3	1	1	3	3	3
3.14 global spontaneity	4	3	2	1	3	1	1	3	1	1
Total	91	59	37	29	67	41	38	71	49	42
**UMSARS**										
Part I	37	–	–	26	–	–	26	–	–	26
Part II	33	26	24	21	26	20	19	31	24	22
Part IV	5	4	4	4	4	4	4	4	4	4
**TUG**										
Steps	95	70	50	42	54	37	45	71	48	43
Time	105	90	41	32	51	32	28	58	33	29

MDS-UPDRS III, the Movement Disorder Society Sponsored Revision of the Unified Parkinson’s Disease Rating Scale part III; UMSARS, the Unified Multiple Systems Atrophy Rating Scale; TUG, Timed Up and Go test.

The patient was able to walk independently after surgery, showed extensities clinician perceived improvement in her facial expressivity, the volume of speech, and speed of gait and turning, and gained 5 kg of weight. Compared with the preoperative condition, by the combined stimulation at the 8-month postoperative follow-up the MDS-UPDRS-III, New Freezing of Gait Questionnaire (NFOGQ), Gait and Fall Questionnaire (GFQ), and Parkinson’s Disease Questionnaire (PDQ-39) scores were improved by 53.8% (91 vs. 42), 57.7% (26 vs. 11), 52.4% (21 vs. 10), and 65.2% (89 vs. 31), respectively.

The double-blind evaluation of the MDS-UPDRS III and TUG was held at the 4- and 8-month postoperative follow-ups, the outcomes are summarized in Table 1 and Figure 2, and the stimulation parameters of DBS and SCS are summarized in Table 2. The combined stimulation consistently provided a better improvement than either DBS or SCS stimulation alone. She achieved 55 and 46% improvement in the MDS-UPDRS-III by DBS stimulation alone at 4- and 8-month postoperative follow-ups, respectively, while 26 and 22% improvement by SCS stimulation alone, respectively, and the improvement of SCS stimulation was mainly embodied in lower-limb symptoms. The patient emphasized that she achieved better postural stability by combined stimulation than either DBS or SCS stimulation alone, especially during the standing up from squatting position. No adverse effects or complications were observed.

**TABLE 2:** The stimulation parameters of DBS and SCS at discharge and 4- and 8-month postoperative.

	Active contacts	Pulse widths (μs)	Frequency (Hz)	Amplitudes (V)
**DBS stimulation**				
discharge	Left STN: C + 9−	60	160	2.0
	Right STN:C + 2−	60	160	1.7
4-month	Left STN: C + 9−	60	160	2.5
	Right STN: C + 2−	60	160	2.0
8-month	Left STN:C + 9−	60	160	2.8
	Right STN: C + 2−	60	160	2.1
**SCS stimulation**				
discharge	4 + 9−10−15 +	210	60	1.05
4-month	4 + 9−10−15 +	210	60	1.05
8-month	4 + 9−10−15 +	210	60	1.05

DBS, deep brain stimulation; SCS, spinal cord stimulation.

## Discussion

To the best of our knowledge, this is the first MSA-P case treated with combined therapy of bilateral STN-DBS and SCS. The combined stimulation provided significant improvement of motor function, including rigidity, bradykinesia, gait disorder, freezing episodes, and postural stability, and her neurological status remained stable and disabilities attenuated.

Although DBS is generally not recommended for patients with MSA, small patient series have reported a mild to significant improvement of dyskinesia, rigidity, and akinesia (Tarsy et al., 2003; Visser-Vandewalle et al., 2003; Meissner et al., 2016). The effect declines over time, but at long-term follow-up the patients’ degree of motor dysfunction that may be more severe in patients with MSA-P was still better with DBS stimulation than without (Visser-Vandewalle et al., 2003). For our patient, severe rigidity and bradykinesia was the main complaints. Our patient achieved 55 and 46% improvement in the MDS-UPDRS-III by DBS stimulation alone at 4 and 8 months follow-up, respectively. Throughout the follow-up period, she did not encounter side effects of dysarthria or dysphagia, which may counteract DBS benefits or lead to pneumonia or pulmonary embolism. Because of overlapping clinical features, patients with MSA-P may present symptoms very similar to PD (Krismer and Wenning, 2017), and intraoperative micro-recording of STN disclosed similar neuronal firing rate and pattern between patients with MSA-P and PD (Berciano et al., 2002; Tarsy et al., 2003; Lezcano et al., 2004). The positive effect of STN stimulation on motor function (most pronounced for rigidity and akinesia) of MSA might be explained by a mechanism similar to that in PD (Neumann et al., 2017). High-frequency stimulation of the STN could suppress elevated beta activity, as intraoperative recordings in the STN (Supplementary Figure 1), and modulate the pathological oscillatory activity in the motor cortex/basal ganglia network (Guridi and Alegre, 2017), thereby inducing clinical benefits.

Besides severe rigidity and bradykinesia, this patient with MSA-P was admitted to our hospital with another main complaint: levodopa-resistant FOG. We have reported that low-thoracic SCS could improve levodopa-resistant FOG in a patient with MSA-P (38% improvement in the gait-related items in the MDS-UPDRS-III) (Zhang et al., 2020), but his rigidity and akinesia symptoms remained unchanged after SCS. Olivia Samotus et al. reported that the key longitudinal outcome of SCS was the reduction in FOG frequency, which was mostly reflected in the FOG-related scales rather than in the MDS-UPDRS-III (Samotus et al., 2020a). Thus, we considered combined stimulation of STN-DBS and SCS for the treatment of this patient with MSA-P with severe levodopa-unresponsive rigidity/bradykinesia and FOG.

Recently, some studies demonstrated that SCS improved gait-associated problems in patients with PD (Agari and Date, 2012; de Andrade et al., 2016; de Souza et al., 2017; Samotus et al., 2018, 2020a), MSA-P (Zhang et al., 2020) or PSP (Samotus et al., 2020b), but the exact neurophysiological mechanisms have not been elucidated. According to basic understanding, hyperactive oscillations of basal ganglia cause brain network dysfunction and interfere with descending input to spinal networks, and affect the normal functional capacity of spinal networks, thereby causing gait impairments (Zhong et al., 2019). Thus, Erich Talamoni Fonoff et al. suggest that SCS mediates its gait effects by modulating ascending afferents and long propriospinal fibers located next to the gray matter of the dorsal horn that reach the brainstem, cerebellum, basal ganglia, and cortical areas (Fonoff et al., 2019). However, there is no compelling evidence to attribute gait effects to simply a supraspinal influence.

Complex and dynamic behavior (e.g., gait) requires the coordination of multiple neural networks to drive. The outcomes of the double-blind evaluation at all follow-ups showed that in the SCS-on/DBS-off condition the improvement was mainly embodied in lower-limb symptoms, and it could be significantly enhanced by SCS-on/DBS-on stimulation. These present observations cannot exclude the likelihood that low thoracic SCS improves the gait-associated problems possibly *via* direct modulation of spinal locomotor networks, the neuromodulation may act in a manner that interrupts the abnormal signals descending from the brain. By targeting the intrinsic spinal networks, in conjunction with rigidity/bradykinesia control using DBS. The combined DBS and SCS stimulation could achieve an integrated effect *via* simultaneous modulation of both the supraspinal circuits and the spinal locomotor networks. Thus, one could have better control over the severe rigidity and akinesia in the lower limbs and gait dysfunction alongside severe FOG.

In conclusion, the combined treatment of bilateral STN-DBS and SCS was an effective symptomatic treatment for severe parkinsonian symptoms and gait-associated problems in our patient with MSA-P. However, this preliminary clinical study needs a longer follow-up to verify clinical improvement and further validation by more cases.

## Data availability statement

The original contributions presented in the study are included in the article/Supplementary material, further inquiries can be directed to the corresponding author/s.

## Ethics statement

The studies involving human participants were reviewed and approved by Ethics Committee of Xuanwu Hospital of Capital Medical University. The patients/participants provided their written informed consent to participate in this study. Written informed consent was obtained from the individual(s) for the publication of any potentially identifiable images or data included in this article.

## Author contributions

JPL and YZ: conceptualization ideas and surgery. SM: clinical assessment. XJ and JLL: collecting data. JPL and SM: data analysis and drafting the manuscript. JPL: visualization. SM, EX, and WM: diagnosis. XZ, YW, and YZ: critical comments. All authors contributed to the article and approved the submitted version.

## Supplementary material

Supplementary Figure 1 Power spectrum density of local field potential recordings showed elevated beta activity (frequency at 21–30 Hz) in the left **(A)** and right **(B)** subthalamic nucleus.

Supplementary Figure 2 The protocol of double-blind phase I trials, the stimulation settings were blinded to the patient, and assessor.

## Abbreviations

MSA-P: multiple system atrophy with predominant parkinsonism
DBS: deep brain stimulation
SCS: spinal cord stimulation
PD: Parkinson’s disease
PSP: progressive supranuclear palsy
STN: subthalamic nucleus
FOG: freezing of gait
MMSE: Mini-Mental State Examination
MoCA: Montreal Cognitive Assessment
HAMA: Hamilton Anxiety Scale
HAMD: Hamilton depression scale
MDS-UPDRS: Movement Disorder Society Sponsored Revision of the Unified Parkinson’s Disease Rating Scale
MRI: magnetic resonance imaging
FDG-PET: ^18^F-fluorodeoxyglucose-positron emission tomography
VMAT2: vesicular monoamine transporter type-2
UMSARS: Unified Multiple Systems Atrophy Rating Scale
TUG: Timed Up and Go
NFOGQ: New Freezing of Gait Questionnaire
GFQ: Gait and Fall Questionnaire
PDQ-39: Parkinson’s Disease Questionnaire.
