# Supplementary figures and images for: Case report: Combined therapy of bilateral subthalamic nucleus deep brain stimulation and spinal cord stimulation significantly improves motor function in a patient with multiple system atrophy with predominant parkinsonism
Source: Front Neurosci. 2022 Aug 1;16:929273. doi: 10.3389/fnins.2022.929273 (PMC9376352; doi:10.3389/fnins.2022.929273)

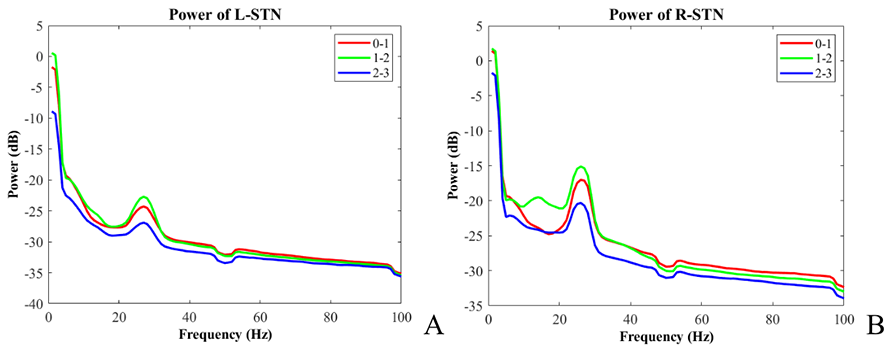

Supplement: Supplementary Figure 1 — Power spectrum density of local field potential recordings showed elevated beta activity (frequency at 21–30 Hz) in the left (A) and right (B) subthalamic nucleus. [file Image_1.tif]

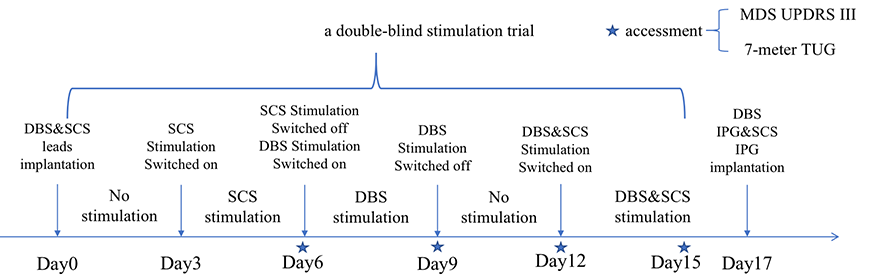

Supplement: Supplementary Figure 2 — The protocol of double-blind phase I trials, the stimulation settings were blinded to the patient, and assessor. [file Image_2.TIF]
